# South Korean geriatrics on Beers Criteria medications at risk of adverse drug events

**DOI:** 10.1371/journal.pone.0191376

**Published:** 2018-03-15

**Authors:** Grace Juyun Kim, Kye Hwa Lee, Ju Han Kim

**Affiliations:** 1 Seoul National University Biomedical Informatics (SNUBI), Division of Biomedical Informatics, Seoul National University College of Medicine, Seoul, Korea; 2 Precision Medicine Center, Seoul National University Hospital, Seoul, Korea; Taipei Veterans General Hospital, TAIWAN

## Abstract

**Background:**

The Beers Criteria released by the American Geriatrics Society includes a list of drugs to avoid in the geriatric population and is frequently used as a safety resource in geriatric pharmacotherapy.

**Objective:**

To evaluate the exposure of South Korean geriatrics to potentially inappropriate medications according to the Beers Criteria and the risk of adverse events from these medications.

**Methods:**

This study included medications recommended to be avoided in patients 65 years or older regardless of concomitant drug therapy or disease. The exposure of South Korean geriatrics to each of the study medications were examined using health claims data of 2011. The number of South Korean geriatrics at risk of experiencing adverse drug events from the study medications were estimated by multiplying the number of patients exposed to the medication in 2011 and the incident rate of the event obtained from literature sources.

**Results:**

This study examined 166,822 geriatrics for Beers Criteria medication exposure and adverse drug event risk. The most prevalent Beers Criteria medication prescribed in South Korean geriatrics >1 day was chlorpheniramine (53.9%) and the adverse drug event with the highest number of this geriatric population at risk of was amitriptyline related dry mouth (4.9%). The proportion of South Korean geriatrics on chronic Beers Criteria medications >1 day at risk of adverse drug events from these medications was significantly higher than in US geriatrics (0.005 vs. 0.001, 2-way ANOVA post hoc pairwise *t*-test *P*<0.0001).

**Conclusions:**

In 2011, over half of South Korean geriatrics was exposed to medications recommended to be avoided in geriatrics and their adverse drug event risk warrants close monitoring of their occurrence.

## Introduction

The geriatric population is at risk for drug related adverse events as they tend to have acute illnesses and are exposed to several medications [1, 2]. A previous study revealed that around half of older populations take five or more medications [2]. This population also has physiological changes attributed to aging and this may influence the pharmacokinetics and pharmacodynamics of drugs increasing the risk of drug therapy [3, 4]. There were studies showing that inappropriate medication prescribing was common in the geriatric population with rates up to 40.0% [3]. Inappropriate prescribing are those practices where the risk of adverse drug events (ADEs) from prescribing the medication is higher than the benefit [3].

This type of inappropriate prescribing is known to be associated with ADEs and hospitalization and around 12% of elderly hospital admissions are caused by adverse drug reactions [5]. ADEs are a significant problem as these increase the morbidity and mortality of patients, and in Western countries, make up 3–5% of hospital admissions and around 10% of hospitalization costs [6].

Beers et al. made criteria with drugs considered inappropriate in the elderly in 1991 [7]. This criteria lists drugs or drug classes to avoid in patients aged 65 years or more due to risk of ADEs including anticholinergic effects, physical dependence, cognitive impairment as well as those that have drug-disease interactions that worsen the disease of the geriatric patient [5]. The goal of the criteria was to improve geriatric pharmacotherapy through decreasing the exposure of the elderly to potentially inappropriate medications. These medications were seen to be commonly prescribed in hospitals and were known to decrease the health of the elderly [6, 7]. This criteria was updated in year 2003, 2012, and 2015 and is now frequently used as a safety resource in geriatric pharmacotherapy, education, and research [8].

The aim of this study was to assess the exposure of South Korean geriatrics to potentially inappropriate medications in the Beers Criteria and their risk of ADEs from the medications. This was performed to measure the extent of ADE risk that South Korean geriatric patients were exposed to and the need of safety measures to prevent the ADEs.

## Materials and methods

### Study population

This was a cross-sectional study including South Korean patients 65 years of age or older. The claims data of this population issued in year 2011 was used to extract the medication use of these patients (claims dataset serial number, HIRA-2011-0133). The dataset used was collected by the Health Insurance Review and Assessment service (HIRA) and includes the health claims data for 3% of the total South Korean population. This population was selected in the dataset using stratified sampling using gender and 5-year age group and was shown to be representative of 95% of the total South Korean population [9, 10]. The total number of patients in this dataset was 1,375,842 and the number of geriatrics 65 years of age or older was 166,822 (12.13%).

### Study medications

The 2015 Beers Criteria medication (BCM) list was used for this study. BCMs to be avoided in patients 65 years or older were included for analysis regardless of concomitant disease or meds. The reason for this was because the health claims data did not provide sufficient information to extract patients who satisfy when to avoid the BCM considering concomitant disease or drug therapy of the patient. The number of medications in the 2015 BCM list was 115 and among these, the number of medications that were to be avoided in the elderly over 65 years of age regardless of concomitant disease or drug therapy was 82.

### Population exposure to study medication

The exposure of the South Korean geriatric study patients to each of the 82 BCMs was examined by counting the number of patients who had a claim for each BCM with a prescription duration of more than one day according to the 2011 HIRA claims data. The count of patients on the med was denoted as *N_med_*. For comparison of South Korean geriatric exposure to BCM with that of US geriatrics, the Part D Prescriber National Summary Report, Calendar Year 2014 was downloaded from the Centers for Medicare & Medicaid Services website. This data included the number of Medicare Part D beneficiaries who had claims to medications [11]. The total number of Medicare beneficiaries in 2014 was 54,095,565 and this was used to estimate the proportion of US geriatrics aged 65 years or older exposed to BCMs [12]. The number of South Koreans on a BCM for one day or less was examined separately to account for ADEs that may have occurred from or short term meds. The number of patients on BCMs for this duration were denoted as *N_med,short_*.

**Fig 1:**
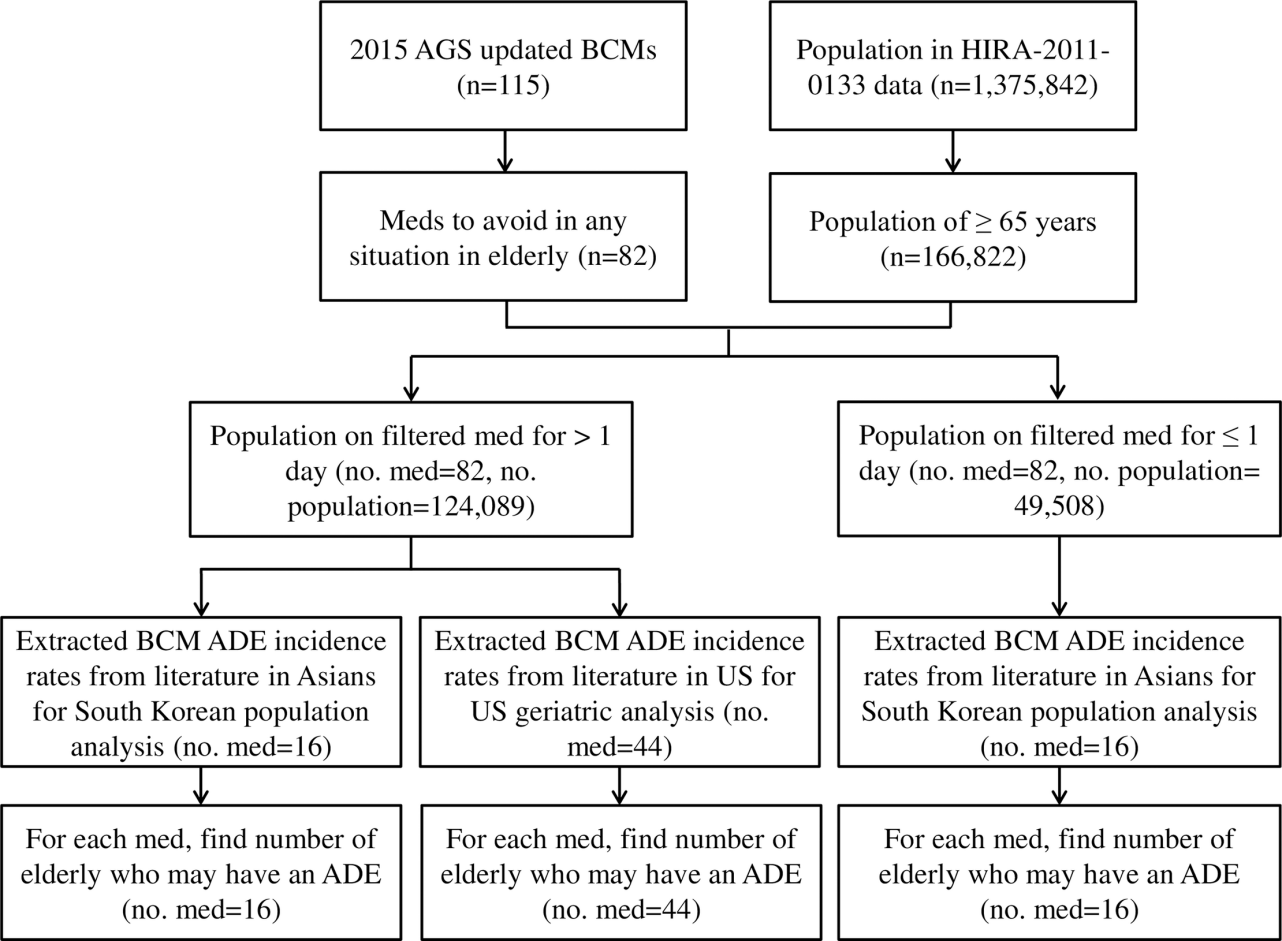
Overall workflow of the study. *AGS* American Geriatrics Society; *ADE* adverse drug event, *BCM* Beers Criteria medication.

The overall workflow for this study is in Fig 1.

### Population at risk of ADE

A literature search in PubMed was performed to extract the incident rate of adverse drug events (ADEs) from the study meds in Asians and the US population for calculating the number of populations at risk of ADEs in South Korea and US, respectively. The literature sources preferred for analysis were those in English or translated in English, included subjects of age 65 years and older, studied subjects of Asian race (or subjects in the US if obtaining rates for US population), and were a meta-analysis. If there was no literature source for the ADE incident rate exclusively in geriatrics 65 years or over, studies including subjects aged <65 years as well as those ≥65 years if available were used. In the case were there were no meta-analyses, single studies were included. If there were multiple studies satisfying the above criteria, the more recent study was used to extract the ADE incident rate. The PubMed search term was “study drug AND elderly (or geriatric) AND side effect (or adverse event) AND Asia” for extracting ADE rates in the South Korean population and “study drug AND elderly (or geriatric) AND side effect (or adverse event)” for extracting ADE rates in the US population. Using the number of exposed South Korean geriatrics to each study med and the med-related ADE incident rates extracted from the literature, the number of geriatrics at risk of the BCM related ADE was calculated. The formula for this calculation was

$$N_{med–ADE}=N_{med}\times Pr(ADE|med),$$ (1)

where *med* indicates the BCM of our study, *N_med–ADE_* the number of geriatrics at risk of an ADE from a *med*, *N_med_* the number of geriatrics exposed to a *med*, and Pr(*ADE*|*med*) the incidence rate of ADE from the *med* in geriatrics.

### Statistical analysis

The Chi-squared test was used to evaluate the difference in frequency of BCMs prescribed in South Korean geriatrics for >1 day versus ≤1 day or versus the BCM prescription frequency in US geriatrics. The proportions of South Koreans on BCMs >1 day versus ≤1 day at risk of ADEs grouped by Medical Dictionary for Regulatory Activities (MedDRA) system organ class (SOC) was compared using 2-way ANOVA using the factors prescription duration and MedDRA SOC, with pairwise *t*-test for post hoc analysis. In addition, 2-way ANOVA was used to compare the proportions of South Koreans on BCMs >1 day and US geriatrics at risk of ADEs again grouped by MedDRA SOC using two factors, the national location of the patients and the SOCs, with pairwise *t*-test for post hoc analysis. The South Korean geriatrics on BCMs for >1 day were used for ADE risk comparison with US geriatrics due to the data source used for US geriatrics including prescription data of chronic disease Medicare beneficiaries [11, 13]. The mean risk of ADEs grouped by SOCs were compared between South Korean geriatrics on BCMs >1 day and ≤1 day as well as South Korean geriatrics on BCMs for >1 day and US geriatrics using the two sample *t*-test. Each of the BCM ADEs were grouped into SOCs using the BioPortal ontology library [14]. All statistical tests were carried out with a significance level of *P*<0.05 using R version 3.3.2 [15].

### Ethical approval

The personal identification information of samples in the South Korean HIRA service and the CMS Part D Prescriber National Summary Report, Calendar Year 2014 was removed prior to data download for this study. Therefore, formal consent of the study sample was not required.

## Results

### Study population

The demographics of the study population are in Table 1. The number of the target geriatric population who were ≥65 years of age was 166,822. The mean (standard deviation, SD) age of the study population was 73.1 (6.5) years and 98,714 (59.2%) were women. The mean (SD) number of medications prescribed per patient was 30 (20.1). The number of people prescribed at least one prescription for a BCM inappropriate in the elderly of 65 years of age or older regardless of concomitant disease or drug was 128,749 (77.2%). The number of people prescribed two or more of such prescriptions was 107,430 (64.4%) and three or more was 91,427 (54.8%).

**Table 1:** Demographics of the study population.

Variable	Total (n = 166,822)
Age, y (mean±SD)	73.1±6.5
Female gender, n (%)	98,714 (59.2)
Medications per person, n (mean±SD)	30±20.1
Population prescribed at least X BCM prescriptions, n (%)	
1	128,749 (77.2)
2	107,430 (64.4)
3	91,427 (54.8)

*SD* standard deviation, *BCM* Beers Criteria medication.

### Population exposure to study medications

The prevalence of South Koreans on BCMs is shown in Table 2. The meds were sorted by prevalence of prescribing BCMs >1 day in South Korean geriatrics. This prevalence was compared with that of US geriatrics. The most prevalent BCM prescribed in South Korean geriatrics for >1 day was chlorpheniramine (53.9%), a first-generation antihistamine, followed by the benzodiazepines, diazepam (23.7%), and alprazolam (13.0%). The most prevalent BCM prescribed in South Korean geriatrics for ≤1 day was chlorpheniramine (21.9%), a first-generation antihistamine, followed by a benzodiazepine, diazepam (4.6%), and ketorolac, a nonsteroidal antiinflammatory drug (3.4%). In US geriatrics, alprazolam (4.7%) was most prevalently prescribed, followed by lorazepam (4.0%), and zolpidem (3.9%). Out of the 82 BCMs, 35 were prescribed at least once in South Korean geriatrics for >1 day and 33 prescribed for ≤1 day while 66 were prescribed in US geriatrics. Comparing prescription rates between the South Korean geriatrics on >1 day or ≤1 day of BCMs, 31 meds were prescribed at a significantly higher rate in South Korean geriatrics prescribed BCMs >1 day, 2 meds at a significantly higher rate in geriatrics prescribed BCMs ≤1 day, and 2 meds were unknown as the prescribing frequency was not available for at least one of the population groups. Comparing prescription rates between the two countries, 22 meds were prescribed at a significantly higher rate in South Korean geriatrics prescribed BCMs >1 day, 35 meds at a significantly higher rate in US geriatrics, and 16 were unknown as the prescribing frequency was not available in the geriatric population of one or either countries.

**Table 2:** Exposure of South Korean and US geriatrics to Beers Criteria medications not recommended in geriatrics regardless of concomitant disease or drugs.

BCM (n = 82)	No. South Korean sample population (%, total sample no. 166,822)		Minimum no. US patients on Medicare Part D on BCM in 2014 (%, total no. 54,095,565)
	On BCM for >1 day	On BCM for ≤1 day	
Chlorpheniramine^✝^	89,923 (53.9)	36,474 (21.9)	NA
Diazepam^✝^^,^*	39,467 (23.7)	7,659 (4.6)	1,077,677 (2.0)
Alprazolam^✝^^,^*	21,705 (13.0)	1,605 (1.0)	2,544,993 (4.7)
Dimenhydrinate^✝^^,^*	19,419 (11.6)	1,990 (1.2)	27 (0.0)
Hydroxyzine^✝^^,^*	17,719 (10.6)	1,406 (0.8)	478,644 (0.9)
Zolpidem^✝^^,^*	14,278 (8.6)	1,856 (1.1)	2,134,655 (3.9)
Orphenadrine^✝^^,^*	11,271 (6.8)	571 (0.3)	48,876 (0.1)
Amitriptyline^✝^^,^*	10,913 (6.5)	551 (0.3)	805,092 (1.5)
Methocarbamol^✝^^,^*	6,747 (4.0)	1,565 (0.9)	376,405 (0.7)
Lorazepam^✝^^,^*	6,375 (3.8)	2,237 (1.3)	2,139,238 (4.0)
Triazolam^✝^^,^*	4,625 (2.8)	497 (0.3)	45,044 (0.1)
Nifedipine^✝^^,^*	3,779 (2.3)	1,255 (0.8)	528,204 (1.0)
Clonazepam^✝^^,^*	3,471 (2.1)	204 (0.1)	1,488,470 (2.8)
Ketorolac^✝^^,^*	1,878 (1.1)	5,594 (3.4)	543,586 (1.0)
Megestrol^✝^^,^*	1,775 (1.1)	327 (0.2)	263,577 (0.5)
Paroxetine^✝^^,^*	1,343 (0.8)	39 (0.0)	852,918 (1.6)
Cyclobenzaprine^✝^^,^*	1,335 (0.8)	67 (0.0)	1,296,038 (2.4)
Imipramine^✝^^,^*	1,307 (0.8)	51 (0.0)	67,450 (0.1)
Phenobarbital^✝^^,^*	889 (0.5)	105 (0.1)	77,327 (0.1)
Clemastine^✝^^,^*	771 (0.5)	83 (0.0)	3,457 (0.0)
Chlordiazepoxide^✝^^,^*	743 (0.4)	63 (0.0)	30,182 (0.1)
Benztropine (oral) ^✝^^,^*	743 (0.4)	51 (0.0)	306,664 (0.6)
Ticlopidine^✝^^,^*	713 (0.4)	32 (0.0)	3,375 (0.0)
Flurazepam^✝^^,^*	632 (0.4)	120 (0.1)	18,289 (0.0)
Clidinium-Chlordiazepoxide^✝^	418 (0.3)	27 (0.0)	NA
Trihexyphenidyl^✝^^,^*	398 (0.2)	22 (0.0)	47,580 (0.1)
Clorazepate^✝^^,^*	365 (0.2)	14 (0.0)	70,224 (0.1)
Triprolidine^✝^	301 (0.2)	23 (0.0)	NA
Doxylamine^✝^	212 (0.1)	17 (0.0)	NA
Atropine (excludes ophthalmic) ^✝^^,^*	182 (0.1)	3,542 (2.1)	368,693 (0.7)
Clomipramine^✝^^,^*	86 (0.1)	1 (0.0)	17,988 (0.0)
Amoxapine^✝^^,^*	20 (0.0)	1 (0.0)	2,099 (0.0)
Pentazocine^✝^	10 (0.0)	2 (0.0)	5,483 (0.0)
Dipyridamole (oral, short-acting)*	4 (0.0)	0 (0)	135,045 (0.2)
Pentobarbital	1 (0.0)	0 (0)	NA
Amobarbital	0 (0)	0 (0)	NA
Brompheniramine	0 (0)	0 (0)	NA
Dexbrompheniramine	0 (0)	0 (0)	NA
Dexchlorpheniramine	0 (0)	0 (0)	NA
Guanabenz	0 (0)	0 (0)	NA
Isoxsuprine	0 (0)	0 (0)	NA
Mephobarbital	0 (0)	0 (0)	NA
Mineral oil, given orally	0 (0)	0 (0)	NA
Quazepam	0 (0)	0 (0)	NA
Meclizine*	0 (0)	0 (0)	1,069,961 (2.0)
Temazepam*	0 (0)	0 (0)	714,706 (1.3)
Promethazine*	0 (0)	0 (0)	695,648 (1.3)
Dicyclomine*	0 (0)	0 (0)	535,628 (1.0)
Glyburide*	0 (0)	0 (0)	346,562 (0.6)
Nortriptyline*	0 (0)	0 (0)	324,594 (0.6)
Carisoprodol*	0 (0)	0 (0)	287,184 (0.5)
Indomethacin*	0 (0)	0 (0)	193,060 (0.4)
Eszopiclone*	0 (0)	0 (0)	113,047 (0.2)
Scopolamine*	0 (0)	0 (0)	101,220 (0.2)
Metaxalone*	0 (0)	0 (0)	80,983 (0.1)
Zaleplon*	0 (0)	0 (0)	72,811 (0.1)
Cyproheptadine*	0 (0)	0 (0)	63,651 (0.1)
Desiccated thyroid*	0 (0)	0 (0)	51,167 (0.1)
Chlorzoxazone*	0 (0)	0 (0)	30,151 (0.1)
Guanfacine*	0 (0)	0 (0)	29,973 (0.1)
Butalbital*	0 (0)	0 (0)	29,554 (0.1)
Hyoscyamine*	0 (0)	0 (0)	24,939 (0.0)
Oxazepam*	0 (0)	0 (0)	21,717 (0.0)
Desipramine*	0 (0)	0 (0)	20,551 (0.0)
Estazolam*	0 (0)	0 (0)	16,951 (0.0)
Diphenhydramine (oral)*	0 (0)	0 (0)	16,007 (0.0)
Methyldopa*	0 (0)	0 (0)	13,878 (0.0)
Meperidine*	0 (0)	0 (0)	13,119 (0.0)
Disopyramide*	0 (0)	0 (0)	4,729 (0.0)
Carbinoxamine*	0 (0)	0 (0)	4,192 (0.0)
Meprobamate*	0 (0)	0 (0)	3,595 (0.0)
Protriptyline*	0 (0)	0 (0)	2,340 (0.0)
Propantheline	0 (0)	0 (0)	1,058 (0.0)
Chlorpropamide	0 (0)	0 (0)	634 (0.0)
Belladonna alkaloids	0 (0)	0 (0)	565 (0.0)
Ergoloid mesylates	0 (0)	0 (0)	500 (0.0)
Trimipramine	0 (0)	0 (0)	323 (0.0)
Butabarbital	0 (0)	0 (0)	283 (0.0)
Secobarbital	0 (0)	0 (0)	61 (0.0)
Reserpine	0 (0)	0 (0)	11 (0.0)
Doxepin >6 mg/d	NA	NA	NA
Insulin, sliding scale	NA	NA	NA

*NA* not available, *BCM* Beers Criteria medication

*Med prescribing frequency is significantly different between South Korean geriatrics on BCM >1 day and US geriatrics according to Chi-square test (*P*<0.05).

✝Med prescribing frequency is significantly different between South Korean geriatrics on BCM >1 day and ≤1 day according to Chi-square test (*P*<0.05).

### Population at risk of ADE from study medications

We estimated the number as well as proportion of geriatrics at risk of ADEs using the incidence rate of ADEs from BCMs in the literature and the exposure of the geriatrics to the BCMs. The number of study BCMs with ADE rates available in the literature was 16 out of 82 for Asians and 44 out of 82 for the US population. The ADE incidence rates and the number of South Korean patients at risk of the ADEs calculated using Eq (1) are in Table 3 and of the US patients in Table 4. The BCMs and ADEs in Tables 3 and 4 were sorted by the number of geriatrics at risk of the ADE. Specifically, for Table 3 the BCMs and ADEs were sorted by the number of South Korean geriatrics on BCMs >1 day at risk of ADEs. The BCM-ADE pair with the highest number of South Koreans on BCMs >1 day at risk of its occurrence was amitriptyline related dry mouth (n = 8,185, 4.9%) followed by amitriptyline related sleepiness (n = 7,508, 4.5%). In addition, dizziness and constipation from amitriptyline and rash/urticaria/pruritus, dizziness/somnolence, dyspnea, and nausea/vomiting from diazepam were among the ten most frequent ADEs that were predicted occur in this South Korean geriatric population on BCMs for for more than 1 day. The BCM-ADE pair with the highest number of South Koreans on BCMs ≤1 day at risk of its occurrence was diazepam related rash/urticaria/pruritus (n = 1,081, 0.6%). This was followed by lorazepam related dizziness/somnolence (n = 1,040, 0.6%), diazepam related dizziness/somnolence (n = 1,021, 0.6%), and diazepam related dyspnea (n = 873, 0.5%). The ADE from BCMs that the highest number of US geriatrics was at risk of was cyclobenzaprine related somnolence (n = 1,296,038, 2.4%). This was followed by cyclobenzaprine related dry mouth (n = 751,702, 1.4%), dicyclomine related dizziness/blurring of vision/dry mouth (n = 368,512, 0.7%), cyclobenzaprine related headache (n = 349,930, 0.6%), and lorazepam related restlessness (n = 320,886, 0.6%).

**Table 3:** Rate and number of the geriatric population in South Korea at risk of adverse drug events from Beers Criteria medications (80 BCM-ADE pairs).

BCM (n = 16)	ADE (n = 56)	Incidence of ADE in Asians (%)	No. South Korean geriatrics with risk of ADE (total no. 166,822)		SOC of ADE (per MedDRA ontology)	Reference of ADE incidence
			on BCM for >1 day	on BCM for ≤1 day		
Amitriptyline	Dry mouth	75	8,185	413	Gastrointestinal disorders	[16]
Amitriptyline	Sleepiness	68.8	7,508	379	Psychiatric disorders	[16]
Diazepam	Rash/urticaria/pruritus	14.1	5,569	1,081	Skin and subcutaneous tissue disorders/immune system disorders/skin and subcutaneous tissue disorders	[17]
Diazepam	Dizziness/somnolence	13.3	5,261	1,021	Nervous system disorders/psychiatric disorders	[17]
Dimenhydrinate	Weakness	26	5,049	517	General disorders and administration site conditions	[18]
Diazepam	Dyspnea	11.4	4,499	873	Cardiac disorders	[17]
Lorazepam	Dizziness/somnolence	46.5	2,964	1,040	Nervous system disorders/psychiatric disorders	[17]
Amitriptyline	Dizziness	25	2,728	138	Nervous system disorders	[16]
Diazepam	Nausea/vomiting	6.3	2,467	479	Gastrointestinal disorders	[17]
Amitriptyline	Constipation	18.8	2,052	104	Gastrointestinal disorders	[16]
Lorazepam	Hypotension	27.1	1,728	606	Vascular disorders	[17]
Lorazepam	Dyspnea	26.3	1,678	589	Cardiac disorders	[17]
Dimenhydrinate	Dizziness	8	1,554	159	Nervous system disorders	[18]
Lorazepam	Rash/urticaria/ pruritus	22.6	1,439	505	Skin and subcutaneous tissue disorders/immune system disorders/skin and subcutaneous tissue disorders	[17]
Clonazepam	Drowsiness	36.8	1,277	75	Psychiatric disorders	[19]
Lorazepam	Nausea/vomiting	14.2	902	317	Gastrointestinal disorders	[17]
Amitriptyline	Palpitations	6.3	688	35	Cardiac disorders	[16]
Amitriptyline	Malaise	6.3	688	35	General disorders and administration site conditions	[16]
Diazepam	Hypotension	1.4	560	109	Vascular disorders	[17]
Dimenhydrinate	Drowsiness	2	388	40	Psychiatric disorders	[18]
Zolpidem	Impaired balance/falls	1.8	257	33	Nervous system disorders/injury, poisoning and procedural complications	[20]
Nifedipine	Mild headache	6.7	253	84	Nervous system disorders	[21]
Zolpidem	Morning drowsiness	1.3	186	24	Psychiatric disorders	[20]
Clonazepam	Dizziness	5.3	184	11	Nervous system disorders	[19]
Phenobarbital	Weight gain	14.7	131	15	Investigations	[22]
Paroxetine	Loss of appetite	8.7	117	3	Metabolism and nutrition disorders	[23]
Paroxetine	Nausea and vomiting	8.7	117	3	Gastrointestinal disorders	[23]
Zolpidem	Amnesia	0.8	114	15	Nervous system disorders	[20]
Zolpidem	Agitation/confusion/ somnambulism	0.7	100	13	Nervous system disorders/psychiatric disorders/nervous system disorders	[20]
Phenobarbital	Nausea, vomiting	10.3	92	11	Gastrointestinal disorders	[22]
Paroxetine	Dry mouth	6.5	87	3	Gastrointestinal disorders	[23]
Paroxetine	Sweating	6.5	87	3	General disorders and administration site conditions	[23]
Zolpidem	Twilight state	0.5	71	9	Nervous system disorders	[23]
Paroxetine	Dizziness	4.3	58	2	Nervous system disorders	[20]
Zolpidem	Dizziness	0.4	57	7	Nervous system disorders	[20]
Zolpidem	Dependence	0.4	57	7	Psychiatric disorders	[20]
Paroxetine	Weight gain	2.2	30	1	Investigations	[23]
Paroxetine	Blurred vision	2.2	30	1	Nervous system disorders	[23]
Zolpidem	Headache	0.2	29	4	Nervous system disorders	[20]
Zolpidem	Nightmare	0.2	29	4	Psychiatric disorders	[20]
Zolpidem	Malaise	0.2	29	4	General disorders and administration site conditions	[20]
Zolpidem	Weakness	0.2	29	4	General disorders and administration site conditions	[20]
Ticlopidine	Cerebral infarction	2.5	18	1	Nervous system disorders	[24]
Zolpidem	Dysgeusia	0.1	14	2	Nervous system disorders	[20]
Phenobarbital	Hepatitis	1.5	13	2	Hepatobiliary disorders	[22]
Ticlopidine	Transient ischemic attack	0.5	4	0	Vascular disorders	[24]
Ticlopidine	Angina pectoris	0.4	3	0	Coronary artery disorders	[24]
Ticlopidine	Peripheral arterial occlusion	0.1	1	0	Vascular disorders	[24]
Estazolam	Falls	16.1	0	0	Injury, poisoning and procedural complications	[25]
Eszopiclone	Dysgeusia	16.2	0	0	Nervous system disorders	[26]
Eszopiclone	Somnolence	5.9	0	0	Psychiatric disorders	[26]
Eszopiclone	Dizziness	2.9	0	0	Nervous system disorders	[26]
Eszopiclone	Dermatitis contact	2.9	0	0	Injury, poisoning and procedural complications	[26]
Eszopiclone	Feeling abnormal	4.3	0	0	General disorders and administration site conditions	[26]
Isoxsuprine	Decreased arterial pressure	22.2	0	0	Investigations	[27]
Isoxsuprine	Headache	19.4	0	0	Nervous system disorders	[27]
Isoxsuprine	Trembling	8.3	0	0	Nervous system disorders	[27]
Isoxsuprine	Nervousness	11.1	0	0	Psychiatric disorders	[27]
Isoxsuprine	Gastrointestinal problems	25.0	0	0	Gastrointestinal disorders	[27]
Isoxsuprine	Skin rash	11.1	0	0	Skin and subcutaneous tissue disorders	[27]
Isoxsuprine	Facial redness	11.1	0	0	Skin and subcutaneous tissue disorders	[27]
Isoxsuprine	Tachycardia	5.6	0	0	Cardiac disorders	[27]
Meperidine	Shivering	9.1	0	0	Musculoskeletal and connective tissue disorders	[28]
Meperidine	Nausea	21.2	0	0	Gastrointestinal disorders	[28]
Meperidine	Pruritus	3.0	0	0	Skin and subcutaneous tissue disorders	[28]
Nortriptyline	Dysarthria	36.8	0	0	Psychiatric disorders	[29]
Nortriptyline	Orthostatic dizziness	42.1	0	0	Nervous system disorders	[29]
Nortriptyline	Sleepiness/sedation	47.4	0	0	Psychiatric disorders/nervous system disorders	[29]
Nortriptyline	Accommodation disturbance	36.8	0	0	Eye disorders	[29]
Nortriptyline	Reducded salivation	60.5	0	0	Gastrointestinal disorders	[29]
Nortriptyline	Diarrhea	23.7	0	0	Gastrointestinal disorders	[29]
Nortriptyline	Constipation	50.0	0	0	Gastrointestinal disorders	[29]
Nortriptyline	Micturition disturbance	39.5	0	0	Renal and urinary disorders	[29]
Nortriptyline	Nausea/vomiting	15.8	0	0	Gastrointestinal disorders	[29]
Nortriptyline	Weight gain	39.5	0	0	Investigations	[29]
Nortriptyline	Weight loss	10.5	0	0	Investigations	[29]
Nortriptyline	Diminished sexual desire	36.8	0	0	Reproductive system and breast disorders	[29]
Doxepin >6 mg/d	Somnolence	14.2	NA	NA	Psychiatric disorders	[30]
Doxepin >6 mg/d	Nervousness	2.9	NA	NA	Psychiatric disorders	[30]

*NA* not available, *BCM* Beers Criteria medication.

**Table 4:** Rate and number of the geriatric population in the US at risk of adverse drug events from Beers Criteria medications (159 BCM-ADE pairs).

BCM (n = 44)	ADE (n = 104)	Incidence of ADE in US (%)	No. US geriatrics with risk of ADE (total no. 54,095,565)	SOC of ADE (per MedDRA ontology)	Reference of ADE incidence
Cyclobenzaprine	Somnolence	100	1,296,038	Psychiatric disorders	[31]
Cyclobenzaprine	Dry mouth	58	751,702	Gastrointestinal disorders	[31]
Dicyclomine	Dizziness/blurring of vision/dry mouth	68.8	368,512	Nervous system disorders/nervous system disorders/gastrointestinal disorders	[32]
Cyclobenzaprine	Headache	27	349,930	Nervous system disorders	[31]
Lorazepam	restlessness	15	320,886	Nervous system disorders	[33]
Atropine (excludes ophthalmic)	Fatigue	84.6	311,914	General disorders and administration site conditions	[34]
Cyclobenzaprine	Dizziness	19	246,247	Nervous system disorders	[31]
Atropine (excludes ophthalmic)	Dyspnea	53.8	198,357	Cardiac disorders	[34]
Benztropine (oral)	Dry mouth	63	193,198	Gastrointestinal disorders	[35]
Cyclobenzaprine	Blurred vision	12	155,525	Nervous system disorders	[31]
Benztropine (oral)	Blurred vision	42	128,799	Nervous system disorders	[35]
Atropine (excludes ophthalmic)	Dry mouth	34.6	127,568	Gastrointestinal disorders	[34]
Clonazepam	Fatigue	8	119,078	General disorders and administration site conditions	[36]
Cyclobenzaprine	Dry throat	8	103,683	Gastrointestinal disorders	[31]
Cyclobenzaprine	Nausea	8	103,683	Gastrointestinal disorders	[31]
Paroxetine	Hyponatremia	12	102,350	Metabolism and nutrition disorders	[37]
Clonazepam	Hypotonia	6	89,308	Musculoskeletal and connective tissue disorders	[36]
Benztropine (oral)	Decreased motor activity	26	79,733	Nervous system disorders	[35]
Benztropine (oral)	Dizziness	26	79,733	Nervous system disorders	[35]
Benztropine (oral)	Drowsiness	24	73,599	Psychiatric disorders	[35]
Benztropine (oral)	Anorexia	20	61,333	Metabolism and nutrition disorders	[35]
Imipramine	Constipation	65	43,843	Gastrointestinal disorders	[38]
Atropine (excludes ophthalmic)	Angina	11.5	42,400	Cardiac disorders	[34]
Benztropine (oral)	Nausea	13	39,866	Gastrointestinal disorders	[35]
Cyproheptadine	Sedation	62.5	39,782	Nervous system disorders	[39]
Nifedipine	Edema	7.5	39,615	Metabolism and nutrition disorders	[40]
Triazolam	Next-day memory impairment/amnesia	83.3	37,522	Nervous system disorders	[41]
Imipramine	Dry mouth	55	37,098	Gastrointestinal disorders	[38]
Zolpidem	Nonvertebral fracture	1.7	36,289	Injury, poisoning and procedural complications	[42]
Cyproheptadine	Dry mouth	56.3	35,836	Gastrointestinal disorders	[39]
Nortriptyline	Sinus tachycardia	10	32,459	Cardiac disorders	[43]
Atropine (excludes ophthalmic)	Palpitations	7.7	28,389	Cardiac disorders	[34]
Imipramine	Tremor	40	26,980	Nervous system disorders	[38]
Imipramine	Drowsiness	40	26,980	Psychiatric disorders	[38]
Diazepam	Headache	2.5	26,942	Nervous system disorders	[44]
Diazepam	Agitation	2.5	26,942	Psychiatric disorders	[44]
Imipramine	Sweating	35	23,608	General disorders and administration site conditions	[38]
Imipramine	Vertigo	35	23,608	Nervous system disorders	[38]
Imipramine	Headache	35	23,608	Nervous system disorders	[38]
Imipramine	Cardiovascular symptoms	35	23,608	Cardiac disorders	[38]
Zolpidem	Hip fracture	1.1	23,481	Injury, poisoning and procedural complications	[42]
Benztropine (oral)	Sweating	7	21,466	General disorders and administration site conditions	[35]
Imipramine	Disturbance of accomodation	30	20,235	Eye disorders	[38]
Ketorolac	Tachycardia	3.5	19,026	Cardiac disorders	[45]
Desipramine	Tiredness	89.5	18,393	General disorders and administration site conditions	[46]
Clorazepate	Drowsiness	26	18,258	Psychiatric disorders	[47]
Nifedipine	Headache	3.4	17,959	Nervous system disorders	[40]
Desipramine	Dry mouth	84.2	17,304	Gastrointestinal disorders	[46]
Desiccated thyroid	Hypertriiodothyroninemia	33.3	17,039	Investigations	[48]
Temazepam	Fatigue/sensation of heaviness/somnolence/eye irritation	2.3	16,367	General disorders and administration site conditions/general disorders and administration site conditions/psychiatric disorders/eye disorders	[49]
Nortriptyline	Intractable constipation	5	16,230	Gastrointestinal disorders	[43]
Nortriptyline	Proarrhythmic event	5	16,230	Cardiac disorders	[43]
Ketorolac	Hypotension	2.8	15,220	Vascular disorders	[45]
Eszopiclone	Unpleasant taste	12.5	14,131	Nervous system disorders	[50]
Nifedipine	Dizziness	2.6	13,733	Nervous system disorders	[40]
Imipramine	Disturbance of micturition	20	13,490	Renal and urinary disorders	[38]
Imipramine	Nausea	20	13,490	Gastrointestinal disorders	[38]
Diazepam	Somnolence	1.2	12,932	Nervous system disorders	[44]
Megestrol	Deep vein thrombosis	4.9	12,915	Vascular disorders	[51]
Cyproheptadine	Dizziness	18.8	11,966	Nervous system disorders	[39]
Cyproheptadine	Nausea and vomiting	18.8	11,966	Gastrointestinal disorders	[39]
Hyoscyamine	Dry mouth/constipation/dizziness/tiredness/headaches/vaginal dryness/night sweats	61.3	11,912	Gastrointestinal disorders/gastrointestinal disorders/nervous system disorders/general disorders and administration site conditions/nervous system disorders/reproductive system and breast disorders/general disorders and administration site conditions	[52]
Ketorolac	Hypertension	2.1	11,415	Vascular disorders	[45]
Ketorolac	Thrombophlebitis	2.1	11,415	Vascular disorders	[45]
Desipramine	Constipation	55.3	11,365	Gastrointestinal disorders	[46]
Glyburide	Edema	3.2	11,090	Metabolism and nutrition disorders	[53]
Imipramine	Sexual dysfunctions	15	10,118	Psychiatric disorders	[38]
Eszopiclone	Dry mouth	8.8	9,948	Gastrointestinal disorders	[50]
Flurazepam	Hangover symptoms	50	9,145	General disorders and administration site conditions	[54]
Dipyridamole (oral short-acting)	Chest pain/headache/nausea/dizziness/pain (not chest)/dyspnea/vomiting/wheezing/syncope/severe hypotension	36	8,766	Cardiac disorders/nervous system disorders/gastrointestinal disorders/nervous system disorders/general disorders and administration site conditions/cardiac disorders/gastrointestinal disorders/respiratory, thoracic and mediastinal disorders/vascular disorders/vascular disorders	[55]
Nifedipine	Constipation	1.6	8,451	Gastrointestinal disorders	[40]
Glyburide	Weight gain	2.4	8,317	Investigations	[53]
Nortriptyline	Persistent myoclonic jerks	2.5	8,115	Nervous system disorders	[43]
Nortriptyline	Severe angina	2.5	8,115	Cardiac disorders	[43]
Nifedipine	Fatigue	1.5	7,923	General disorders and administration site conditions	[40]
Chlordiazepoxide	Drowsy	25.8	7,787	Psychiatric disorders	[56]
Eszopiclone	Dizziness	6.6	7,461	Nervous system disorders	[50]
Eszopiclone	Somnolence	6.6	7,461	Psychiatric disorders	[50]
Guanfacine	Fatigue	23.5	7,044	General disorders and administration site conditions	[57]
Desipramine	Insomnia	34.2	7,028	Psychiatric disorders	[46]
Desipramine	Increased sweating	34.2	7,028	General disorders and administration site conditions	[46]
Clorazepate	Depression	10	7,022	Psychiatric disorders	[47]
Imipramine	Ataxia	10	6,745	General disorders and administration site conditions	[38]
Imipramine	Vomiting	10	6,745	Gastrointestinal disorders	[38]
Eszopiclone	Pain	5.9	6,670	General disorders and administration site conditions	[50]
Diphenhydramine (oral)	Delirium symptoms	41.2	6,595	Psychiatric disorders	[58]
Desipramine	Headache	28.9	5,939	Nervous system disorders	[46]
Desipramine	Lightheadedness	28.9	5,939	Nervous system disorders	[46]
Clomipramine	Dry mouth	32.2	5,792	Gastrointestinal disorders	[59]
Estazolam	Drugged feeling	33.3	5,645	Nervous system disorders	[60]
Nifedipine	Chest pain	1	5,282	Cardiac disorders	[40]
Nifedipine	Flushing	1	5,282	Vascular disorders	[40]
Nifedipine	Abdominal pain	1	5,282	Gastrointestinal disorders	[40]
Nifedipine	Nausea	0.9	4,754	Gastrointestinal disorders	[40]
Eszopiclone	Nervousness	3.7	4,183	Psychiatric disorders	[50]
Eszopiclone	Rash	3.7	4,183	Skin and subcutaneous tissue disorders	[50]
Cyproheptadine	Blurred vision	6.3	4,010	Nervous system disorders	[39]
Ketorolac	Angina pectoris	0.7	3,805	Cardiac disorders	[45]
Ketorolac	Cardiac failure congestive	0.7	3,805	Cardiac disorders	[45]
Ketorolac	Supraventricular tachycardia	0.7	3,805	Cardiac disorders	[45]
Ketorolac	Flushing	0.7	3,805	Vascular disorders	[45]
Imipramine	Increased energy	5	3,373	General disorders and administration site conditions	[38]
Eszopiclone	Accidental injury	2.9	3,278	Injury, poisoning and procedural complications	[50]
Clomipramine	Dizziness	15.9	2,860	Nervous system disorders	[59]
Glyburide	Hypoglycemia	0.8	2,772	Metabolism and nutrition disorders	[53]
Clomipramine	Constipation	14.6	2,626	Gastrointestinal disorders	[59]
Pentazocine	Drowsy/sleepy	47.2	2,588	Psychiatric disorders	[61]
Indomethacin	Cardiovascular and cerebrovascular events	1.33	2,568	Vascular disorders/nervous system disorders	[62]
Eszopiclone	Back pain	2.2	2,487	Musculoskeletal and connective tissue disorders	[50]
Eszopiclone	Peripheral edema	2.2	2,487	Metabolism and nutrition disorders	[50]
Eszopiclone	Arthralgia	2.2	2,487	Musculoskeletal and connective tissue disorders	[50]
Eszopiclone	Anxiety	2.2	2,487	Psychiatric disorders	[50]
Estazolam	Dizziness	13.3	2,254	Nervous system disorders	[60]
Clomipramine	Erectile dysfunction	10.4	1,871	Reproductive system and breast disorders	[59]
Disopyramide	Dry mouth	37	1,750	Gastrointestinal disorders	[63]
Eszopiclone	Emotional lability	1.5	1,696	Psychiatric disorders	[50]
Eszopiclone	Memory impairment	1.5	1,696	Nervous system disorders	[50]
Desipramine	Orthostatic symptoms	7.9	1,624	Vascular disorders	[46]
Desipramine	Palpitations	7.9	1,624	Cardiac disorders	[46]
Amoxapine	Composite of anticholinergic symptoms/cardiovascular/neurological/sedative complaints	68.1	1,429	Nervous system disorders/cardiac disorders	[64]
Clorazepate	Headache	2	1,404	Nervous system disorders	[47]
Disopyramide	Headache	29.6	1,400	Nervous system disorders	[63]
Disopyramide	Bowel changes	29.6	1,400	Gastrointestinal disorders	[63]
Diphenhydramine (oral)	Required new urinary catheter	7.9	1,265	Surgical and medical procedures	[58]
Disopyramide	Urinary complaints	25.9	1,225	Renal and urinary disorders	[63]
Disopyramide	Weakness	22.2	1,050	General disorders and administration site conditions	[63]
Disopyramide	Nausea	22.2	1,050	Gastrointestinal disorders	[63]
Disopyramide	Palpitations	22.2	1,050	Cardiac disorders	[63]
Disopyramide	Lightheadedness	22.2	1,050	Nervous system disorders	[63]
Clomipramine	Insomnia	4.2	755	Psychiatric disorders	[59]
Propantheline	Dry mouth	56.3	596	Gastrointestinal disorders	[65]
Diphenhydramine (oral)	Behavioral disturbance	3.5	560	Psychiatric disorders	[58]
Estazolam	Headache	3.3	559	Nervous system disorders	[60]
Estazolam	Hangover	3.3	559	General disorders and administration site conditions	[60]
Clomipramine	Nervousness	2.9	522	Psychiatric disorders	[59]
Promethazine	Dystonia/extrapyrimidal symptoms/oversedation/delirium/ respiratory depression	0.1	401	Nervous system disorders/nervous system disorders/nervous system disorders/psychiatric disorders/nervous system disorders	[66]
Butalbital	Somnolence	1	296	Psychiatric disorders	[67]
Butalbital	Dizziness	1	296	Nervous system disorders	[67]
Diphenhydramine (oral)	Use of physical restraints	1.8	288	Surgical and medical procedures	[58]
Protriptyline	Dysuria	11.1	260	Renal and urinary disorders	[68]
Protriptyline	Nervousness	11.1	260	Psychiatric disorders	[68]
Pentazocine	Nausea	2.8	154	Gastrointestinal disorders	[61]
Butalbital	Nausea	0.5	148	Gastrointestinal disorders	[67]
Butalbital	Nasopharyngitis	0.5	148	Respiratory, thoracic and mediastinal disorders	[67]
Propantheline	Urinary hesitancy	12.5	132	Renal and urinary disorders	[65]
Butalbital	Paresthesia	0.3	89	Nervous system disorders	[67]
Propantheline	Spastic colon	6.3	67	Gastrointestinal disorders	[65]
Pentazocine	Asleep	0.9	49	Psychiatric disorders	[61]
Ticlopidine	Rash	1	32	Skin and subcutaneous tissue disorders	[69]
Ticlopidine	Thrombocytopenia	0.6	19	Blood and lymphatic system disorders	[69]
Ticlopidine	Bleeding	0.4	13	Vascular disorders	[69]
Ticlopidine	Gastrointestinal	0.4	13	Gastrointestinal disorders	[69]
Ticlopidine	Neutropenia	0.4	13	Blood and lymphatic system disorders	[69]
Clemastine	Life-threatening ventricular arrhythmias	0.1	2	Cardiac disorders	[70]
Chlorpheniramine	Drowsiness/nausea/euphoria	25	NA	Psychiatric disorders/gastrointestinal disorders/psychiatric disorders	[71]
Guanabenz	Dry mouth	37	NA	Gastrointestinal disorders	[72]
Guanabenz	Drowsiness	13	NA	Psychiatric disorders	[72]
Pentobarbital	Hypotension	64.7	NA	Vascular disorders	[73]
Quazepam	Hangover symptoms	54.5	NA	General disorders and administration site conditions	[54]

*NA* not available, *BCM* Beers Criteria medication.

**Fig 2:**
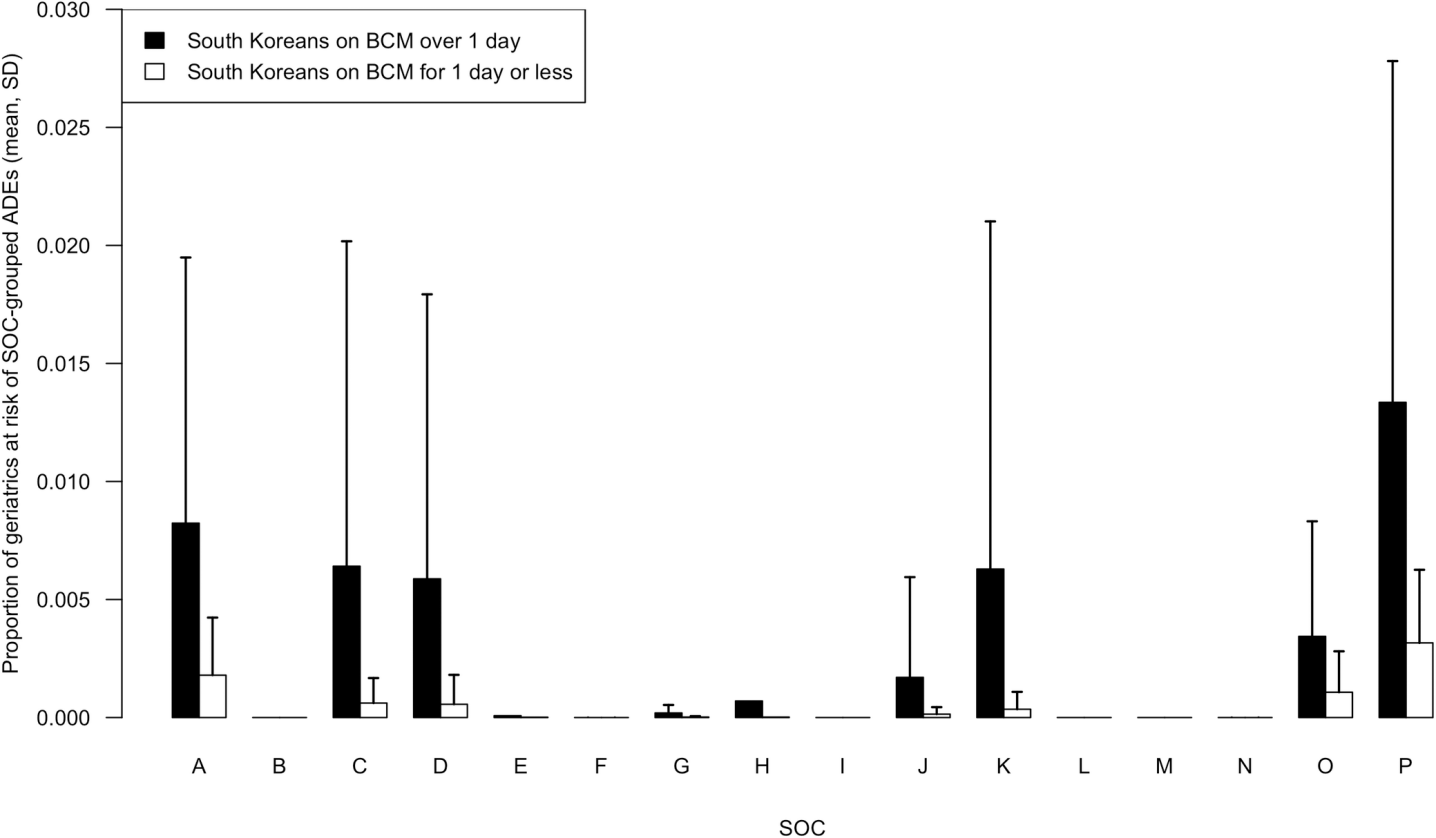
Mean (SD) proportion of South Koreans on Beers Criteria medications >1 day and ≤1 day at risk of Beers Criteria medication related adverse drug events grouped by system organ class. The population number used as the denominator to obtain the proportion in the y-axis was the number of South Korean geriatrics 65 years of age or older, n = 166,822. The SOCs were *A* cardiac disorders (Beers Criteria medication-ADE pairs, n = 5); *B* eye disorders (n = 1); *C* gastrointestinal disorders (n = 13); *D* general disorders and administration site conditions (n = 6); *E* hepatobiliary disorders (n = 1); *F* injury, poisoning and procedural complications (n = 2); *G* investigations (n = 5); *H* metabolism and nutrition disorders (n = 1); *I* musculoskeletal and connective tissue disorders (n = 1); *J* nervous system disorders (n = 18); *K* psychiatric disorders (n = 11); *L* renal and urinary disorders (n = 1); *M* reproductive system and breast disorders (n = 1); *N* skin and subcutaneous tissue disorders (n = 3); *O* vascular disorders (n = 4); *P* composite of multiple SOCs (n = 7). *ADE* adverse drug event; *BCM* Beers Criteria medication; *SOC* system organ class; *SD* standard deviation.

**Fig 3:**
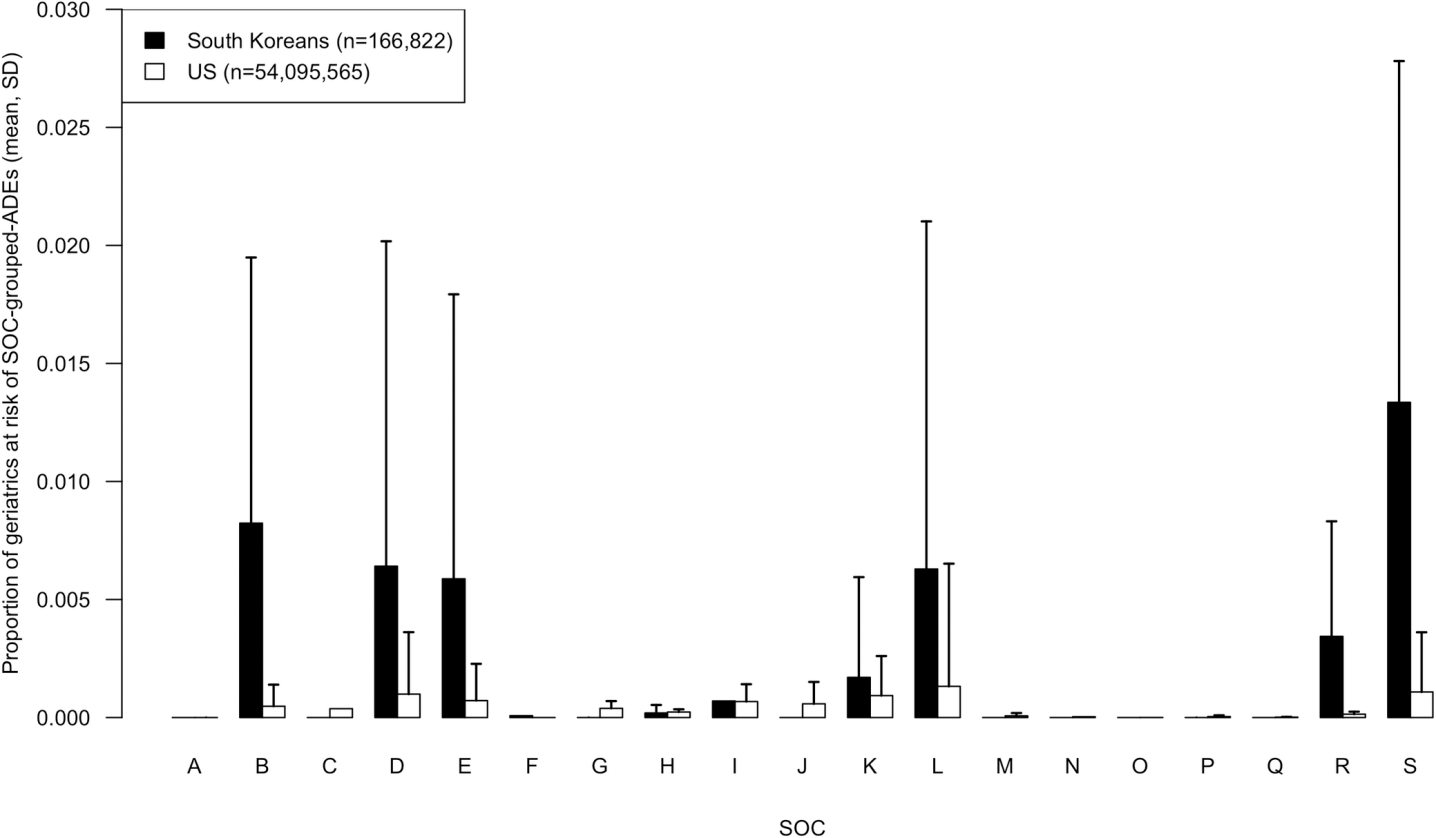
Mean (SD) proportion of South Koreans on Beers Criteria medications >1 day and US geriatrics at risk of Beers Criteria medication related adverse drug events grouped by system organ class. The SOCs were *A* blood and lymphatic system disorders (Beers Criteria medication-ADE pairs, n = 0 for South Korea, n = 2 for US); *B* cardiac disorders (n = 5 for South Korea, n = 15 for US); *C* eye disorders (n = 1 for South Korea, n = 1 for US); *D* gastrointestinal disorders (n = 13 for South Korea, n = 30 for US); *E* general disorders and administration site conditions (n = 6 for South Korea, n = 15 for US); *F* hepatobiliary disorders (n = 1 for South Korea, n = 0 for US); *G* injury, poisoning and procedural complications (n = 2 for South Korea, n = 3 for US); *H* investigations (n = 5 for South Korea, n = 2 for US); *I* metabolism and nutrition disorders (n = 1 for South Korea, n = 6 for US); *J* musculoskeletal and connective tissue disorders (n = 1 for South Korea, n = 3 for US); *K* nervous system disorders (n = 18 for South Korea, n = 33 for US); *L* psychiatric disorders (n = 11 for South Korea, n = 22 for US); *M* renal and urinary disorders (n = 1 for South Korea, n = 4 for US); *N* reproductive system and breast disorders (n = 1 for South Korea, n = 1 for US); *O* respiratory, thoracic and mediastinal disorders (n = 0 for South Korea, n = 1 for US); *P* skin and subcutaneous tissue disorders (n = 3 for South Korea, n = 2 for US); *Q* surgical and medical procedures (n = 0 for South Korea, n = 2 for US); *R* vascular disorders (n = 4 for South Korea, n = 9 for US); *S* composite of multiple SOCs (n = 7 for South Korea, n = 8 for US). *ADE* adverse drug event; *SOC* system organ class; *SD* standard deviation.

The mean proportion of South Korean on BCMs for >1 day versus ≤1 day at risk of ADEs grouped according to MedDRA SOC is in Fig 2. The mean proportion of South Korean on BCMs for >1 day and US geriatrics at risk of ADEs grouped according to MedDRA SOC is in Fig 3. Out of the total 26 SOCs in MedDRA, the BCM ADEs which South Korean geriatrics (regardless of duration of BCM prescription) may be at risk from corresponded to 15 single SOCs and 4 multiple SOC combinations. The 15 single SOCs were “cardiac disorders”, “eye disorders”, “gastrointestinal disorders”, “general disorders and administration site conditions”, “hepatobiliary disorders”, “injury, poisoning and procedural complications”, “investigations”, “metabolism and nutrition disorders”, “Musculoskeletal and connective tissue disorders”, “nervous system disorders”, “psychiatric disorders”, “renal and urinary disorders”, “reproductive system and breast disorders”, “skin and subcutaneous tissue disorders”, and “vascular disorders”. Seven BCM-ADE pairs included ADEs that were a composite of multiple ADEs that were grouped into more than one SOC.

The BCM ADEs which US geriatrics were at risk from corresponded to 17 single SOCs and 8 multiple SOC combinations. The single SOCs were “blood and lymphatic system disorders”, “cardiac disorders”, “eye disorders”, “gastrointestinal disorders”, “general disorders and administration site conditions”, “injury, poisoning and procedural complications”, “investigations”, “metabolism and nutrition disorders”, “musculoskeletal and connective tissue disorders”, “nervous system disorders”, “psychiatric disorders”, “renal and urinary disorders”, “reproductive system and breast disorders”, “respiratory, thoracic and mediastinal disorders”, “skin and subcutaneous tissue disorders”, “surgical and medical procedures”, and “vascular disorders”. Eight BCM-ADE pairs included ADEs that were a composite of multiple ADEs that were grouped into more than one SOC.

The SOC with the BCM related ADE which most South Korean geriatrics on BCMs for >1 day were at risk of was the “composite of multiple SOCs” group (mean proportion = 0.0134). The reason for this was because the third and fourth most common ADEs in this population which were rash/urticaria/pruritus and dizziness/somnolence from diazepam corresponded to multiple adverse events. Excluding the “composite of multiple SOCs” group, this geriatric population was most at risk of ADEs in the “cardiac disorders” SOC (mean proportion = 0.0082). This was followed by the “gastrointestinal disorders” SOC (mean proportion = 0.0064) and “general disorders and administration site conditions” SOC (mean proportion = 0.0059). The SOC with the BCM related ADE which most South Korean geriatrics on BCMs for ≤1 day were at risk of was the “composite of multiple SOCs” group (mean proportion = 0.0032). The reason for this was because the first and second most common ADE in this population which were rash/urticaria/pruritus from diazepam and dizziness/somnolence from lorazepam corresponded to multiple adverse events. Excluding the “composite of multiple SOCs” group, this population was most at risk of ADEs in the “cardiac disorders” SOC (mean proportion = 0.0018) followed by the “vascular disorders” SOC (mean proportion = 0.0011) and “gastrointestinal disorders” SOC (mean proportion = 0.0006).

The SOC with the BCM related ADE which most US geriatrics were at risk of was “psychiatric disorders” (mean proportion = 0.0013). This was because the most common ADE in US geriatrics, cyclobenzaprine related somnolence, corresponded to the SOC “psychiatric disorders”. This was followed by the “composite of multiple SOCs” (mean proportion = 0.0011) and the “gastrointestinal disorders” SOC (mean proportion = 0.0010) and. The SOC including the most BCM-ADE pairs was “nervous system disorders” for both South Koreans on BCMs for more than 1 day and US geriatric cases at 18 pairs and 33 pairs, respectively.

Using the 16 SOCs where South Koreans had ADE rates available, the proportion of South Korean geriatrics prescribed a BCM >1 day versus ≤1 day were compared. The mean overall proportion of South Korean geriatrics on BCMs >1 day at risk of experiencing an ADE of 0.005 was significantly higher than that of South Korean geriatrics on BCMs ≤1 day of 0.001 (2-way ANOVA post hoc pairwise *t*-test, *P* = 0.001). Although not significant, South Korean geriatrics on BCMs >1 day were shown to have a higher mean risk of ADEs grouped into 10 SOCs “cardiac disorders”, gastrointestinal disorders”, “general disorders and administration site conditions”, “hepatobiliary disorders”, “investigations”, “metabolism and nutrition disorders”, “nervous system disorders”, “psychiatric disorders”, “vascular disorders”, and “composite of multiple SOCs” than geriatrics on BCMs ≤1 day. There were no SOCs where South Korean geriatrics on BCMs ≤1 day had a higher mean risk of ADEs than geriatrics on BCMs >1 day. There were no SOCs where South Korean geriatrics on BCMs for 1 day or less were of higher risk than South Korean geriatrics on BCMs >1 day. Comparing the mean risk of SOC-grouped ADEs between SOCs, the mean risk of ADEs grouped into the “composite of multiple SOCs” group was significantly higher than the ADEs grouped into the SOCs “investigations”, “nervous system disorders”, and “skin and subcutaneous tissue disorders” respectively in the South Korean geriatrics on BCMs.

Using the 15 SOCs where both South Korean and US populations had ADE rates available, the proportion of South Korean and US geriatrics at risk of SOC-grouped ADEs from BCMs were also compared. The mean proportion of geriatrics at risk of experiencing the ADEs was significantly higher in South Korean geriatrics on BCMs >1 day at 0.005 compared to US geriatrics at 0.001 (2-way ANOVA post hoc pairwise *t*-test *P*<0.0001). Although not significant, for 8 out of the 15 SOCs where ADEs were grouped into (“cardiac disorders”, “gastrointestinal disorders”, “general disorders and administration site conditions “, “metabolism and nutrition disorders”, “nervous system disorders”, “psychiatric disorders”, “vascular disorders”, “composite of multiple SOCs”), South Korean geriatrics on BCMs >1 day were shown to have a higher mean risk of ADEs than the US geriatrics while the US geriatric population had a higher mean risk of ADEs than the South Korean geriatric population for 7 SOCs which were “eye disorders”, “injury, poisoning and procedural complications”, “investigations”, “musculoskeletal and connective tissue disorders”, “renal and urinary disorders”, “reproductive system and breast disorders”, and “skin and subcutaneous tissue disorders”. Finally, the mean risk of ADEs grouped into the “composite of multiple SOCs” group was significantly higher than the ADEs grouped into the SOCs “cardiac disorders”, “gastrointestinal disorders”, “general disorders and administration site conditions”, “injury, poisoning and procedural complications”, “investigations”, “metabolism and nutrition disorders”, “nervous system disorders”, “psychiatric disorders”, “renal and urinary disorders”, “skin and subcutaneous tissue disorders”, and “vascular disorders” respectively in the South Korean geriatrics on BCMs >1 day and US geriatric populations combined.

## Discussion

This study discovered that the exposure of South Korean geriatrics to BCMs was prevalent in that over half of this population was exposed to these medications and the proportion of the population at risk of the ADEs from BCMs was around three-fold higher in South Korean geriatrics (limited to those prescribed BCMs >1 day) compared to US geriatrics. BCM classes that were most prevalently prescribed in South Korean geriatrics in year 2011 regardless of duration of BCM prescription were first generation antihistamines and benzodiazepines. Specific medications of the first generation antihistamines were chlorpheniramine, dimenhydrinate, and hydroxyzine. These medications have risk of ADEs such as dizziness or drowsiness and this may be debilitating in the elderly as the ADEs may lead to falls or fractures. The benzodiazepines were diazepam and alprazolam and use of these medications in geriatrics increases their risk of cognitive impairment, delirium, falls, fractures, and motor vehicle crashes. The high prescribing rate of these medications in South Korean geriatrics is concerning and this warrants heightened awareness in prescribers regarding the risk of ADEs from BCMs. This issue is becoming more important due to the increased lifespan of the population and use of multiple medications in the geriatric population.

Notable differences in BCM exposure patterns between South Korean geriatrics prescribed chronic (in present study >1 day) and short duration (in present study, 1 day or less) BCMs were that ketorolac and atropine were prescribed more frequently in a short duration of 1 day or less instead of chronically. This may be due to ketorolac being indicated for short term pain or surgical procedures and atropine is indicated acutely for cardiac arrest or organophosphate poisoning [74]. Although other than atropine and ketorolac, the absolute number of South Korean geriatrics prescribed BCMs short term were smaller than geriatrics prescribed BCMs longer term, the relative prescribing trend of the latter BCMs were similar in both populations. The trend of BCM prescribing between South Korea and the US was also similar. The most prevalently prescribed meds for US geriatrics in 2014 were benzodiazepines including alprazolam, lorazepam, zolpidem, and clonazepam. Alprazolam was one of the most commonly prescribed medications in both South Korea and the US.

In South Korean geriatrics prescribed BCMs for >1 day, the ADE rates from amitriptyline were among the highest out of all BCM ADEs examined in this study. Specifically, dry mouth from amitriptyline was the ADE with the highest number of geriatrics at risk of experiencing. Sleepiness, dizziness, and constipation related to amitriptyline were other ADEs that many South Korean geriatrics were at risk of. The reasons for this trend was because the number of patients on amitriptyline and the incidence of ADEs from this drug were high. Diazepam related rash/urticaria/pruritus was the 3^rd^ most common ADE predicted to occur in South Korean geriatrics after amitriptyline ADEs and this also reflects the high number of geriatrics on diazepam. Thus, monitoring geriatrics for anticholinergic changes or toxicities after prescribing medications is important and necessary. The SOCs of ADEs that most of this South Korean population was at risk of were “cardiac disorders” (mean proportion = 0.0082), “gastrointestinal disorders” (mean proportion = 0.0064), and “general disorders and administration site conditions” (mean proportion = 0.0059), after excluding “composite of multiple SOCs” (mean proportion = 0.0134). This shows that monitoring geriatrics for their change in cardiac system or gastrointestinal condition for the possibility of ADEs from medications and adjusting their drug treatment accordingly may improve the safety of drug therapy in geriatrics.

Similarly, for geriatrics of South Korea prescribed BCMs for 1 day or less, ADEs from the benzodiazepines diazepam and lorazepam including rash/urticarial/pruritus, dizziness/somnolence, and dyspnea were those that most of these geriatrics were at risk of. Dry mouth from amitriptyline was also one of the high risk ADEs in this population. Therefore, monitoring geriatrics for their neuropsychiatric, cardiac, and anticholinergic symptoms after medication use and prevention of these ADEs is necessary. Considering that ADEs of the SOC “cardiac disorders” was the most prevalent ADEs this population was at risk of (mean proportion of population at risk = 0.0018) excluding the “composite of multiple SOCs” group, care to avoid medications with cardiotoxicity in this population may be needed.

Examining ADEs predicted in US geriatrics, the trend of ADEs that geriatrics were at risk of were not similar to those in South Korean geriatrics. The ADE that the highest number of US geriatrics was at risk of having was somnolence from cyclobenzaprine. The other ADEs that many US geriatrics were at risk of experiencing were dry mouth, headache, dizziness, and blurred vision from cyclobenzaprine, restlessness from lorazepam, and dizziness/blurring of vision/dry mouth from dicyclomine, and fatigue and dyspnea from atropine (excluding ophthalmic). The most common SOCs of ADEs that US geriatrics were at risk of were “psychiatric disorders” (mean proportion = 0.0013), “gastrointestinal disorders” (mean proportion = 0.0010), and “nervous system disorders” (mean proportion = 0.0009), after excluding “composite of multiple SOCs” (mean proportion = 0.0011).

Comparing the mean proportion of patients at risk of ADEs in 15 SOC groups, South Korean geriatrics on BCMs >1 day were at a higher risk of ADEs in 7 single SOCs plus the “composite of multiple SOCs” group than US geriatrics, although not statistically significant. The 7 single SOCs were “cardiac disorders”, “gastrointestinal disorders”, “general disorders and administration site conditions”, “metabolism and nutrition disorders”, “nervous system disorders”, “psychiatric disorders”, and “vascular disorders”. However, the proportion of geriatrics at risk of the 15 SOC-grouped ADEs combined was statistically significantly higher in South Korea than in the US, showing that medication prescribing for geriatrics in South Korea may require modification or further monitoring regarding its safety outcomes. Although not statistically significant, US geriatrics were at a higher risk of experiencing ADEs in the SOCs “eye disorders”, “injury, poisoning and procedural complications”, “investigations”, “musculoskeletal disorders”, “renal and urinary disorders”, “reproductive system and breast disorders”, and “skin and subcutaneous tissue disorders” than South Korean geriatrics on BCMs >1 day.

This study was the first to systematically examine the exposure of all BCMs in South Korean and US geriatrics and the risk of ADEs from these medications. This analysis enabled a comprehensive overview of the extent of geriatric risk of ADEs using population data. These results may be generalizable to the total national population as the South Korean HIRA national patient sample dataset was shown to represent the total South Korean population [9] and the US Medicare Part D data includes prescription information of American geriatrics 65 years or older [75]. Therefore, the results of this study may be used as a reference to evaluate the current drug therapy in geriatrics.

As this study was cross sectional, there were some limitations. Firstly, the results of this study present medication exposure and ADE risk data for a one-year range providing only a snapshot of BCM exposure and ADE risk. Data of a longer period would enable examination of the change in med exposure or ADE risk over time providing stronger evidence for risk of ADEs from BCMs and enable the inclusion of additional BCMs. The latter is due to the fact that some BCMs were permitted to be used as an alternate to a med used for a certain indication. In this study, as the medication history of a patient was infeasible to determine over a year, we excluded BCMs that were to be avoided under particular medication use or disease histories. Secondly, it was not possible to determine if the patients actually took the drugs as this study used claims data. Thirdly, follow up of patients’ clinical status was not possible. Confirming if the patients on BCMs had an ADE with the claims data would provide direct evidence of ADE risk from the med. However, whether the patients exposed to BCMs experienced ADEs is unknown with our study as the patients in the study data were anonymized and linking the claims data of patients in this study to other data was not possible. Lastly, considering that approximately 0.2% of the South Korean geriatric population are using hospice care, our study may have overestimated the exposure of geriatrics exposed to BCMs as this criteria applies to geriatrics not receiving hospice or palliative care. This population could not be excluded from this study sample because information on whether the geriatric was having this type of care was not discernable from our study data. However, this overestimation did not alter the trend or direction of the study results.

## Conclusions

To conclude, this study found that there is room for improvement in South Korean geriatric drug therapy through enhanced awareness and education of clinicians regarding medications that may be potentially inappropriate for geriatrics. This was known from the fact that at least half of South Korean geriatrics were exposed to medications recommended to be avoided in geriatrics according to the Beers Criteria and a significantly higher proportion of South Korean geriatrics on BCMs >1 day were at risk of ADEs from the BCMs compared to US geriatrics. Heightened awareness from clinicians regarding safe geriatric drug therapy may contribute to increased quality of drug treatment in South Korean geriatrics.
